# Supplementary material for: Spatiotemporal droplet dispersion measurements demonstrate face masks reduce risks from singing
Source: Sci Rep. 2021 Dec 17;11:24183. doi: 10.1038/s41598-021-03519-x (PMC8683488; doi:10.1038/s41598-021-03519-x)

**Supplementary Materials**

**Supplementary Table 1:** Demographic data of participants

**Supplementary Figure 1:** Demonstration of a participant standing in the laser-safe booth (left) and singing whilst the camera took images at 3000 fps whilst illuminated by laser.

**Supplementary Figure 2:** Time resolved transmission of droplets for first four tasks with and without wearing face masks for 16 participants (one participant knocked the laser apparatus when exhaling with a mask)

**Supplementary Figure 3:** Time resolved transmission of droplets for six participants who sang ‘Happy Birthday’ with and without masks (one participant knocked the laser apparatus when singing ‘Happy Birthday’ without a mask)

**Supplementary Video 1:** Video of time vector plots of droplets being produced, after a participant sang the note ‘la’.

**Supplementary Table 1:** Demographic data of participants

|  | N=20 |
| --- | --- |
| Age median (IQR) | 42.0 (27.0) |
| Female n( %) | 14 (70) |
| Ethnicity n (%)  White British  Any Other White Background  Asian/Asian British | 11 (55)  8 (40)  1 (5) |
| Body Mass Index n (%)  18.5 to <25  25 to-<30  ≥30  Missing | 11 (55)  6 (30)  1 (5)  2 (10) |
| Smoking Status  Non-smoker  Ex-smoker | 18 (90)  2 (10) |
| Alcohol Intake  None  Small (1 small drink per day/a couple of pints of beer a week)  Average (1 medium drink per day/up to 7 pints of beer a week) Large: (1 large drink per day/more than 7 pints of beer a week) | 8 (40)  4 (20)  5 (25)  3 (15) |

**Supplementary Figure 1**

1. Participant standing with head against a stop to prevent laser eye damage


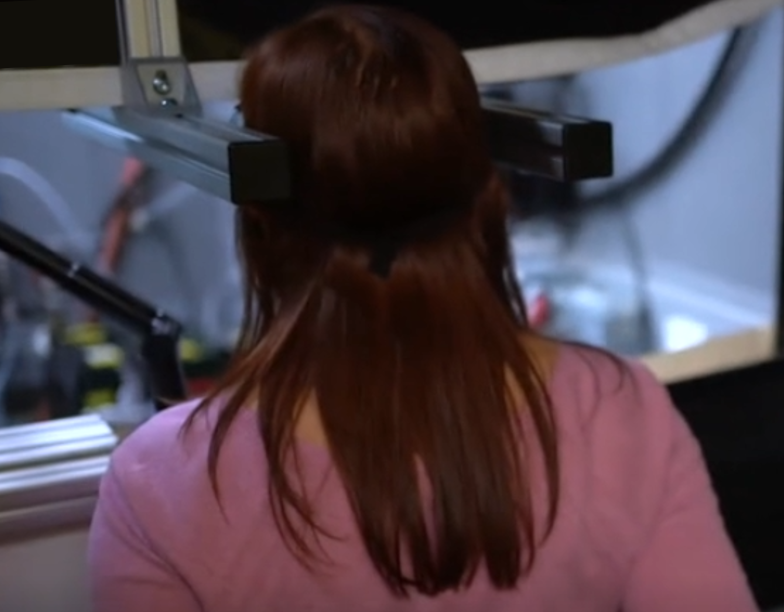


1. Laser safety goggles visible as the participant sings when the laser is on


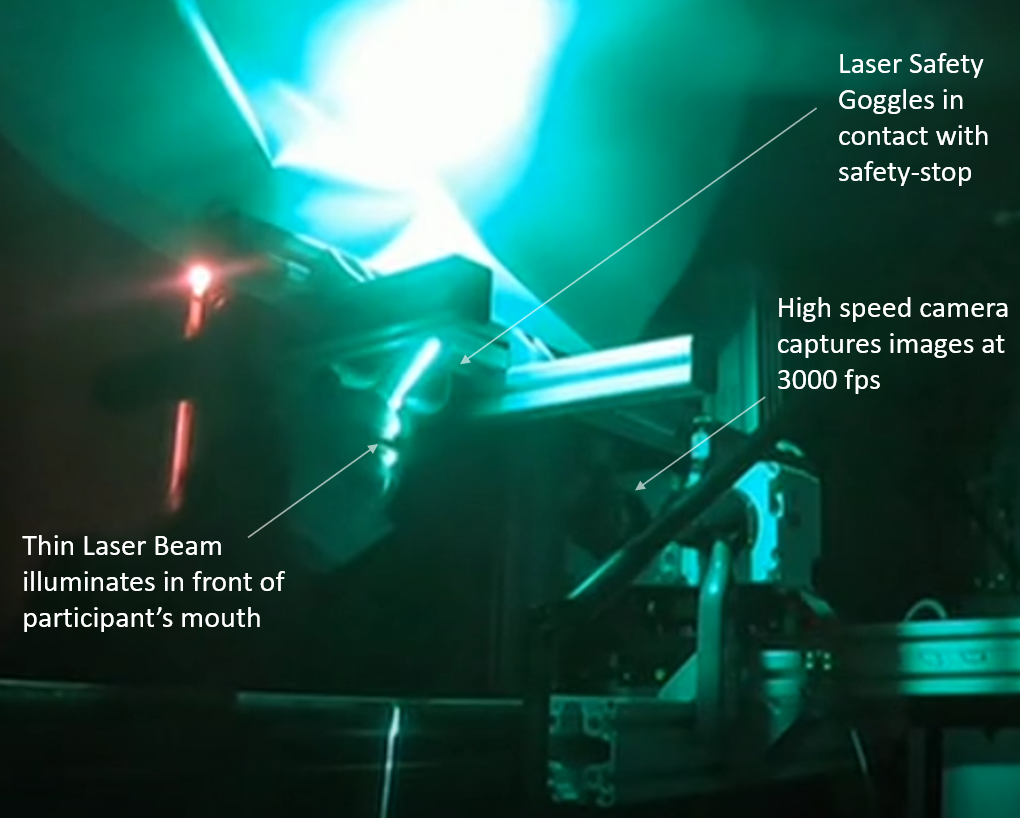


**Supplementary Figure 2:** Time resolved transmission of droplets for all other participants not represented in Figure 2B, when exhaling; saying ‘hello’; saying ‘snake’ and singing the note ‘la’. Again, there was no consistent pattern for number of droplets produced across the various tasks. No result is available for participant 9 for exhaling as the laser was accidentally knocked creating erroneous results and is hence omitted.


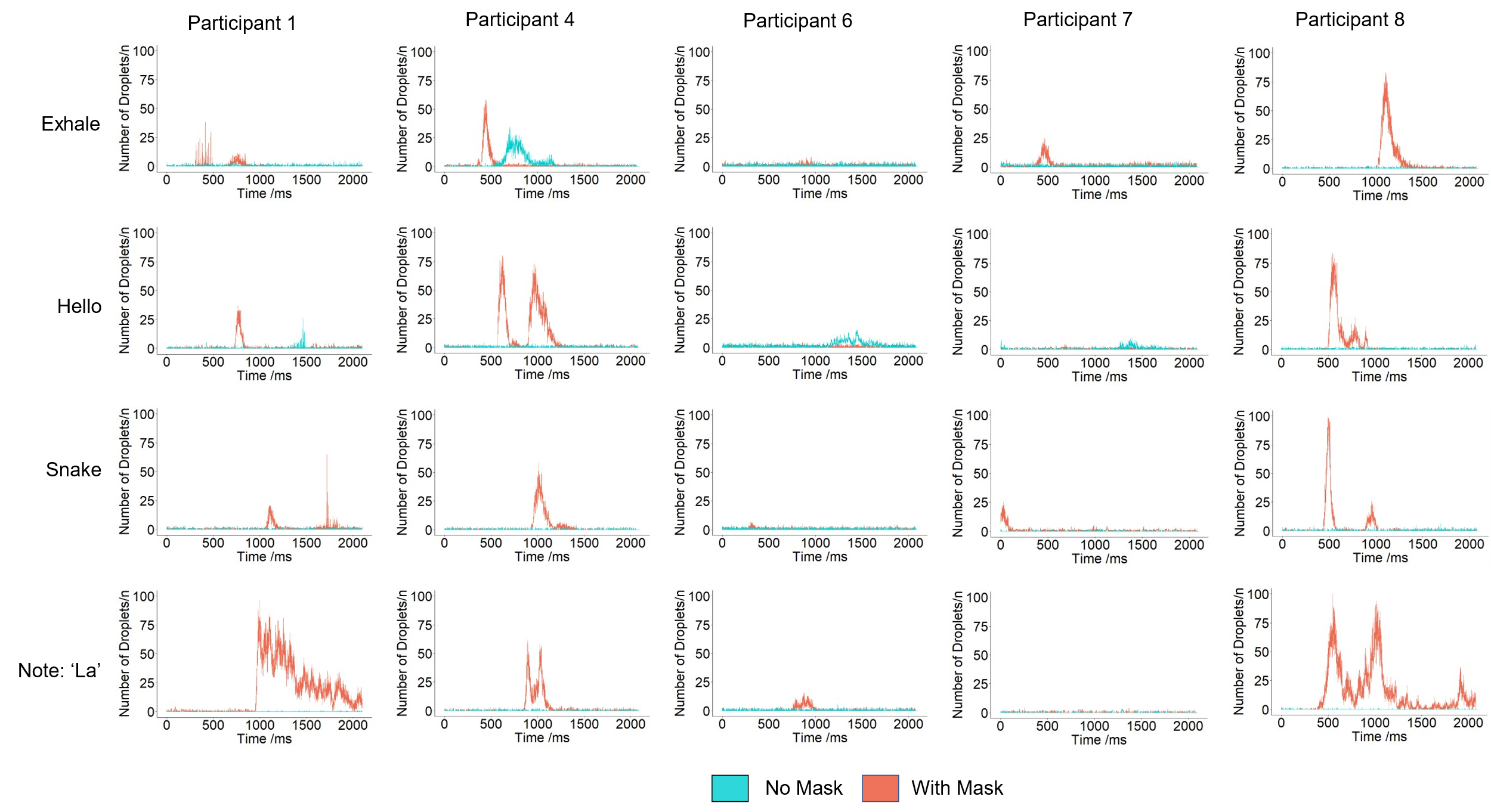


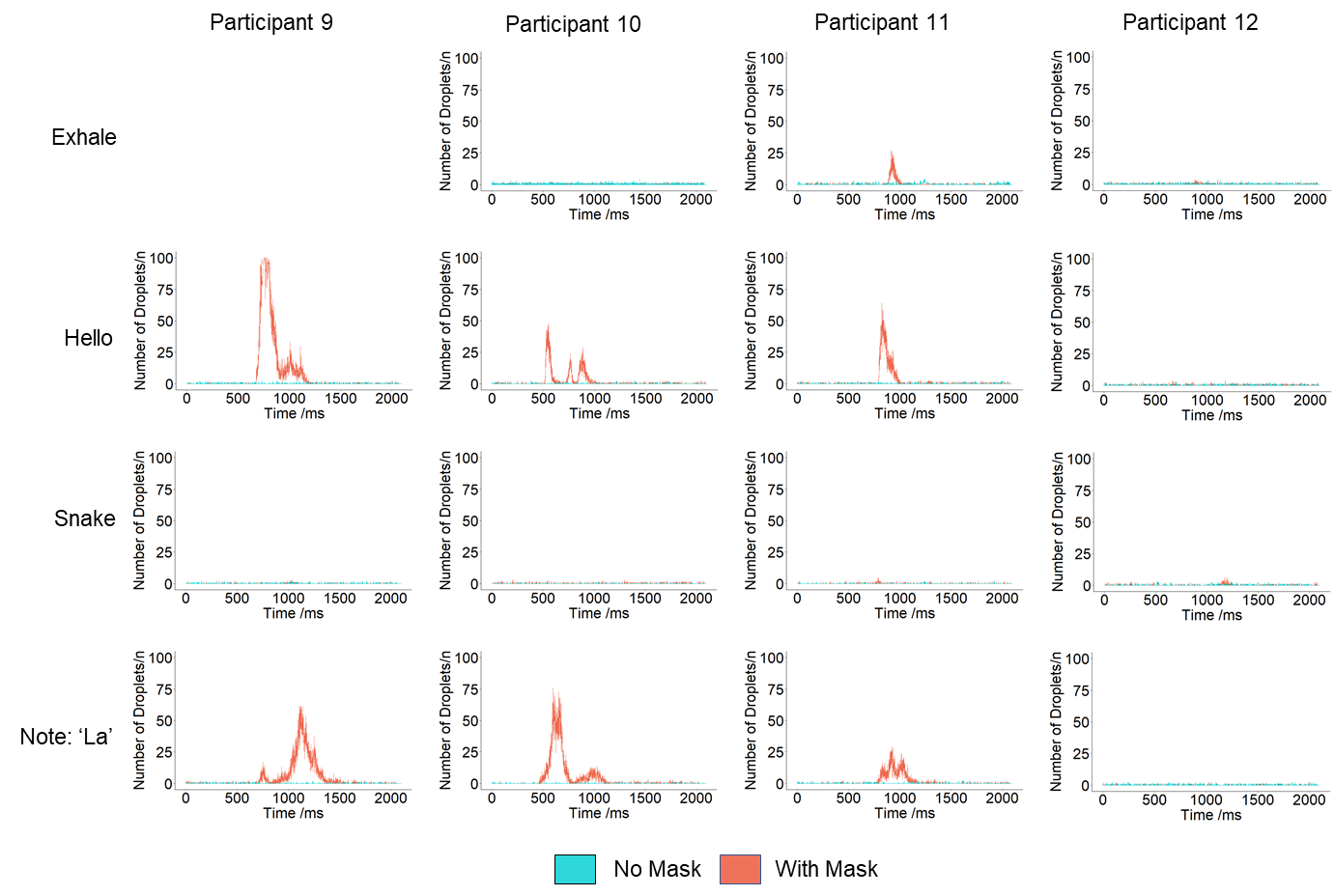


**Supplementary Figure 3:** Time resolved transmission of droplets for 6 participants who sang the first two lines of ‘Happy Birthday’. There is no consistent pattern between individuals as noted with the other tasks. No result is available for participant 16 as the laser was accidentally knocked creating erroneous results and is hence omitted.


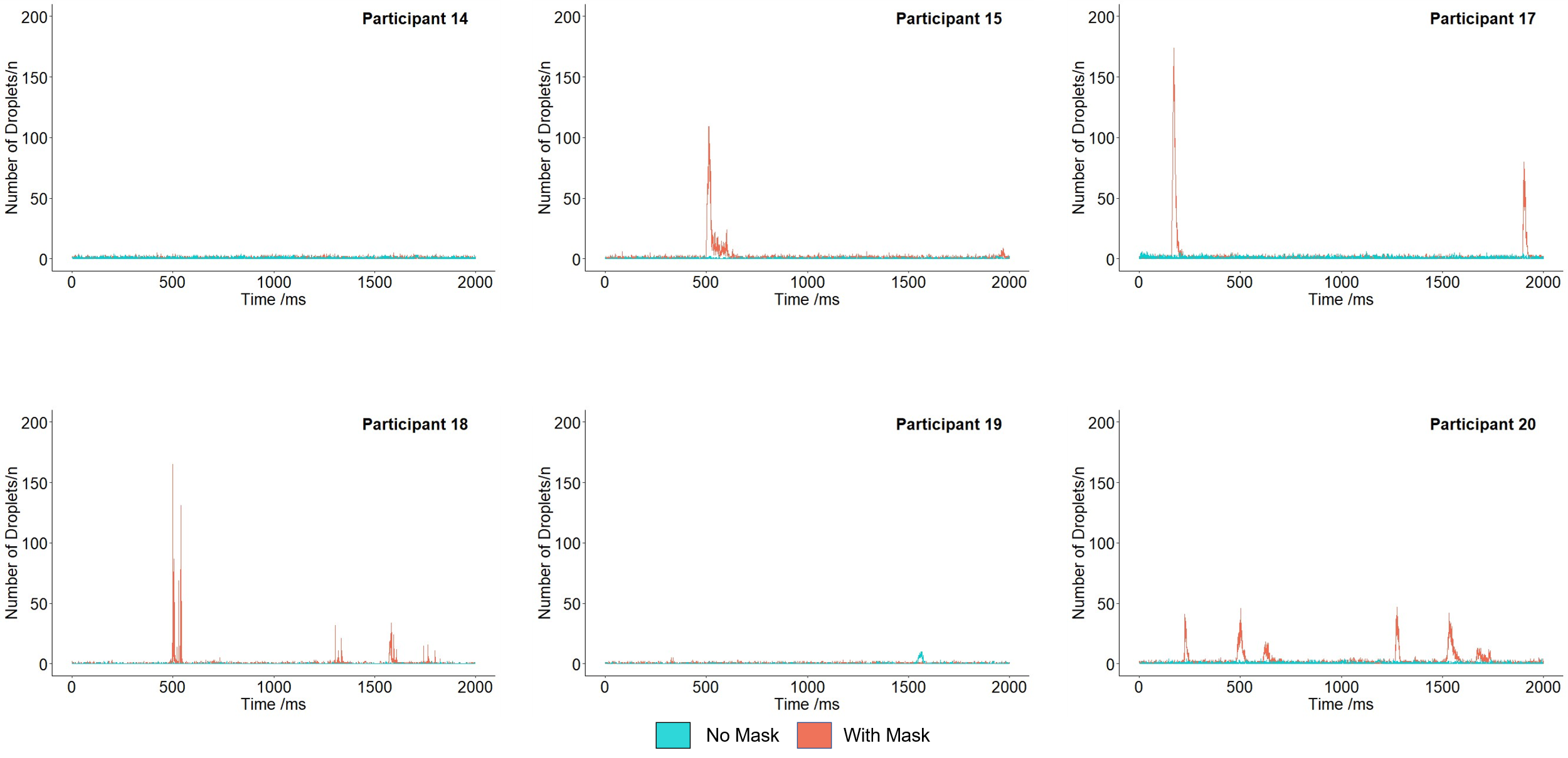

Supplement: Supplementary file 1 — Supplementary Information. [file 41598_2021_3519_MOESM1_ESM.docx]
